# Two-stage topic modelling of scientific publications: A case study of University of Nairobi, Kenya

**DOI:** 10.1371/journal.pone.0243208

**Published:** 2021-01-07

**Authors:** Leacky Muchene, Wende Safari

**Affiliations:** 1 Statistics, StatsDecide Analytics and Consulting Limited, Nairobi, Kenya; 2 MODES, University of A Coruña, A Coruña, Spain; University of Sao Paulo, BRAZIL

## Abstract

Unsupervised statistical analysis of unstructured data has gained wide acceptance especially in natural language processing and text mining domains. Topic modelling with Latent Dirichlet Allocation is one such statistical tool that has been successfully applied to synthesize collections of legal, biomedical documents and journalistic topics. We applied a novel two-stage topic modelling approach and illustrated the methodology with data from a collection of published abstracts from the University of Nairobi, Kenya. In the first stage, topic modelling with Latent Dirichlet Allocation was applied to derive the per-document topic probabilities. To more succinctly present the topics, in the second stage, hierarchical clustering with Hellinger distance was applied to derive the final clusters of topics. The analysis showed that dominant research themes in the university include: HIV and malaria research, research on agricultural and veterinary services as well as cross-cutting themes in humanities and social sciences. Further, the use of hierarchical clustering in the second stage reduces the discovered latent topics to clusters of homogeneous topics.

## Introduction

Natural language processing (NLP) is the ability of machines to read, understand and interpret human language. Over the past decade, NLP has gained popularity especially in artificial intelligence (for instance, chatbots and speech recognition) and human language machine translation. The NLP syntax and semantic analysis methods are based on statistical, probabilistic and machine learning methods [1], thereby attracting considerable interest in academic research, medical research and business intelligence applications. NLP covers a broad range of subtopics such as topic modelling, sentiment analysis, text tagging and language translation. This manuscript’s focus is on latent topic modelling as applied to unstructured text corpora of scientific publications.

In recent past, topic modelling has emerged as an effective tool for discovering useful structure in digital text database resources with significant impact on stimulating research culture in academic research [2–4]. Topic modelling tools provide a fast and feasible way of extracting latent semantic structure in documents and establishing links between the latent topics, a task that would otherwise be time consuming or impossible for large databases. To extract the latent structure, topic models often assume a generative model for each document in the collection (a collection of text documents is referred to as a corpus). The main underlying assumption is then to treat each observed document text as a bag of words derived from a fixed latent number of topics. The topics are then composed of specific words from the corpus vocabulary [5].

Given that a text corpus contains non-negative entries, Non-Negative Matrix Factorization (NMF) can be applied to decompose the high-dimensional corpus into two lower-dimensional matrices: one for the documents grouped by topic (document-topic distribution), while the second contains words comprising the topics (word-topic distribution) [6]. While similar in spirit to NMF, Probabilistic Latent Semantic Analysis (PLSA) posits a probabilistic model rather than Singular Value Decomposition to derive the two matrices (document-topic distribution and word-topic distribution) representation of a text corpus [7]. Latent Dirichlet Allocation (LDA) [8] further extends PLSA by allowing dirichlet priors for both the document-topic distribution and word-topic distribution matrices. Estimation in LDA can then be performed using Gibbs sampling within a Bayesian framework.

While these models assume independence between topics, extensions to allow for direct correlation between topics such as Correlated Topic Models [9], or indirectly via grouped topic models such as Hierarchical Dirichlet Process (HDP) [10] and Group Latent Dirichlet Allocation (GLDA) [11], exist. Further, topic modelling tools addressing the transitional nature of information such as Dynamic Topic Models (DTM) [12] can be used to evaluate the evolution of latent topics over time [13–15]. Note that most of these methods constitute LDA or an extension of LDA. Hence, in principle, LDA is widely adopted in topic modelling.

The basic idea behind LDA is that a document is generated from a finite mixture of topics distribution where each topic is a distribution over words from the vocabulary. By learning the distributions, the most probable words for each topic can then be used to potentially provide human readable topics. Because of its superiority in analysis of large-scale document collections, better results have been obtained in both academia and non-academia areas such as in [16–19].

Attempts at extracting dominant themes from text copora using LDA-based models are not new. For instance, similar to the analysis presented in this manuscript [20–22], applied LDA-based methods to abstracts of scientific publications in order to extract dominant themes. Further [23], applied LDA to New York Times’ coverage of nuclear technology since 1945 and concluded possible temporal variation of the topics. Using LDA [2], obtained potential health topics related to seasonal influenza, allergies, obesity and temporal disease surveillance data as discussed in social media.

However, in Africa, there is a paucity of studies that utilize statistical methodology for unstructured data on collections of documents such as digital repositories [24]. The work presented here seeks to contribute in this domain in the African context. The case study presented in this manuscript is inspired by the growing list of publications from the University of Nairobi’s digital repository. The University of Nairobi is the oldest and largest university in East Africa, with over 500 academic programs and a student population of more than 80000. According to the university website’s “Fact file” page, the university has a 4.5 billion (Kenyan Shilling) annual research kitty. With such research financing, it is expected that a significant number of peer-reviewed academic manuscripts may be published across the university departments. Indeed, on the University’s digital repository, there are over 27000 journal articles and more than 38000 theses and dissertations [25]. The journal articles are further classified into collections under six colleges: College of Agriculture and Veterinary Sciences (CAVS: 5188 documents), College of Architecture and Engineering (CAE: 1376 documents), College of Biological and Physical Sciences (CBPS: 4089 documents), College of Education and External Studies (CEES: 994 documents), College of Health Sciences (CHS: 9609 documents) and College of Humanities and Social Sciences (CHSS: 6342 documents) [26].

The contribution of this manuscript is twofold: first, we showcase some statistical tools for processing unstructured data which is largely encountered in developing countries. For instance, except for donor-funded projects in HIV and Tuberculosis, there are often no proper electronic databases setup for medical records and paper-based records are predominant [27–29]. Further, where available, digital information is often presented as unstructured text on company websites, social media websites such as Twitter or in university e-repositories. Thus, we apply text mining techniques to gather information about scientific publications from the University of Nairobi’s e-repository into a convenient data format and further process the data with open-source R statistical software [30] and associated packages.

The second objective is to perform an unsupervised classification [31] of the University of Nairobi e-repository’s “Journal articles” collection using text mining techniques [32–34]. The goal here is to ultimately have an overview of the predominant research themes at the university. Since the university’s e-repository is subdivided into thematic collections by college, we expect that documents from different colleges can be distinguished by topics, and this division will provide a comprehensive overview of the University’s research topics and potential overlap in research themes between the colleges. To this end, topic modelling avails tools to discover predominant themes in a corpus of documents [2, 8, 21, 23] and allows a document to contribute to multiple themes whereas the natural document classification by college to a large extent presumes colleges are independent strata.

Often, for large text copora, LDA results in a large number of latent topics, say 100 latent topics. Subsequently, succinct presentation and/or visualization of the topic distribution is not trivial. To this end, we propose a two-stage analysis of the case study presented in this manuscript. In the first stage, classical LDA as described in the methodology section (and mostly applied in the related references) is applied to the text copora. For ease of visualization of the original LDA topic composition, we propose simple animation of the topics’ word cloud. Subsequently, the discovered topics are further reduced into clusters of related topics by use of a classical hierarchical clustering algorithm with appropriate topic distance metric. To the best of our knowledge, no previous studies have combined LDA with hierarchical clustering in this manner. The novelty of this manuscript therefore lies in the sequential application of LDA and hierarchical clustering to text copora.

The manuscript is organized as follows. First, we describe the case study data and necessary pre-processing steps performed on it. Thereafter, we provide an explanation of the methodology applied in both determining a reasonable number of topics in the documents to model and the final topic modelling using LDA. The results of model fitting are then provided in addition to a concluding section. Additional relevant output is cited in the manuscript and presented separately as supplementary materials to the manuscript.

## Materials and methods

### Data

Publications available on the University of Nairobi’s “Journal Articles” section of the e-repository (from 1884 to 2019) were downloaded for each of the six colleges separately. A user-defined R function was used to scrape the publications metadata into an R dataframe. Key R packages used for this step include: rvest [35], stringi [36], and tidyverse [37] for website scraping, string processing and general data manipulations, respectively and both foreach [38] and doParallel [39] for parallel execution to speed up the computation burden, where possible. For each publication (hereby referred to as a “document”), the resulting metadata comprised of a title, abstract, authors, publication date amongst other information. For the purpose of this manuscript, only the document abstracts were of interest. We further tagged each document with its source (college: denoting the collection under which it was filed) to later link the discovered topics with the document origin. Only documents with a valid abstract were considered. Hence, duplicate entries or abstracts with less than 50 or more than 400 words after excluding punctuation and English stop words were excluded. The resulting data comprised of three columns containing a unique document identifier, document abstract and the college identifier. Table 1 shows the final sample size across the colleges. The number of documents varied by college although the mean and median abstract length was comparable.

**Table 1:** Summary of available documents per college.

College	n	Percent	Mean	Median
CAE	581	5.38	115	103
CAVS	2560	23.72	112	102
CBPS	1895	17.56	113	101
CEES	306	2.84	122	111
CHSS	1199	11.11	120	107
CHS	4250	39.38	103	93

Percent: (*n*/*M*) * 100 where *n* is the number of documents per college and *M* = 10791. Mean, Median: the mean and median number of words in the processed (no punctuation or stop words) abstracts per college.

### Topic modelling with LDA

Consider the collection of *M* = 10791 documents described in the Data section of this manuscript comprising of a vocabulary of *V* = 9246 unique words. A document is then a collection of *N* words **W** = (*w*_1_, *w*_2_, …, *w*_*N*_) where *w*_*n*_ is the *n*^*th*^ word in the document. Note that the order of occurrence of the words in the original text does not matter (hence the “bag of words” assumption). Subsequently, the final text corpus is a collection of *M* documents denoted by *D* = {**W**_1_, **W**_2_, …, **W**_*M*_}.

LDA considers documents as a finite mixture of latent topics with each topic being defined by a distribution of words from the vocabulary. More formally, to generate a document **W** ∈ *M*:

1. Choose *N* ∼ *Poisson*(*ξ*): denoting the document length—the number of words in the document.
2. Choose *θ* ∼ *Dir*(*α*): the topic distribution for document **W** (across all topics).
3. For each of the *N* words *w*_*n*_:
  (a) Choose a topic *z*_*n*_ ∼ *Multinomial*(*θ*).
  (b) Choose a word *w*_*n*_ from *p*(*w*_*n*_ ∣ *z*_*n*_, *β*): conditional distribution of the word given a topic and words comprising the topic.

Note that *ξ* and *α* are hyperparameters for the corresponding distributions while *β* denotes the distribution of words across the topics. For ease of computation, the number of topics is fixed *a priori*. Optimization of the quantities of interest (topics with their corresponding word probabilities, topic distribution for each word and subsequently, the topic composition for each document) can utilize Bayesian Monte Carlo simulation with Gibbs sampling, Laplace approximation, convexity-based variational inferences amongst others. Readers interested in the technical details of LDA inference are referred to [8, 40], where the LDA methodology is comprehensively covered.

For each topic, a word distribution is derived. Subsequently, for each document, a posterior topic probability is computed (for all topics). The final output from LDA is the posterior word distribution across the topics (often denoted as *β*) and the posterior document topic distribution (often denoted as *γ*).

Critical to LDA is that a document contributes to all latent topics (with varying probability). This is the principal advantage of LDA in the context of document classification (as is the cases study in this manuscript), since it is expected that although a scientific publication may be based on one dominant topic, several latent sub-topics may be present.

On the other hand, the latent topics from LDA are assumed to be independent of each other, which may not be accurate in case of co-occurring topics. For instance, a topic on smoking is more likely to occur together with a topic on lung cancer and less likely to co-occur with a topic on movie reviews. Due to topic independence assumption, LDA would treat smoking and lung cancer themes as independent of each other.

An alternative approach to identifying related topics is to perform Correlated Topics Modelling (CTM) [9] rather than LDA. CTM relaxes the independence assumption of LDA by allowing for potential correlation between topics. However, CTM is much more computationally intensive and our attempt to fit a CTM model with either 50 or 100 correlated topics failed. We instead propose to perform hierarchical clustering [31] of the LDA output for two reasons:

1. To cluster similar topics (based on their document-topic probability). While this is not a replacement for CTM, it may prove useful for identifying related topics without the computational constraints imposed by CTM.
2. To further reduce the dimensions of the discovered topics, thereby, providing concise visualization and synthesis of the results.

To this end, a hierarchichal clustering model with agglomerative clustering [41] is performed using the Hellinger distance [42]. For sensitivity analysis, the cosine distance [43] is used for classification and the resulting clusters compared.

#### Determining the number of topics

To perform topic modelling, one of the critical tuning parameters is the number of topics to model, which is assumed to be known and fixed *a priori*. The user has to specify the number of topics to model with LDA [8, 44], which like any other model tuning parameter can have an influence on the performance of the classification algorithm. Often, when performing document classification, the number of topics may not be known beforehand though we may have an informed guess on the scope of topics the documents cover. For the data presented in this manuscript, we already know that the documents are derived from six (potentially independent) colleges. Within each college, however, there may be further hierarchy in terms of faculties, schools or departments. One of the approaches to determining the number of topics is to assume that these six colleges correspond to six distinct topics. With this approach, the analysis goal is to compare the unsupervised topic modelling results based on six topics to the actual document source [45]. However, LDA considers topics as latent collection of words occurring frequently together and may therefore discover topics or thematic areas not previously considered to be existing in the documents.

Alternatively, when no informative prior knowledge is available on the approximate number of topics in the documents, ldatuning::FindTopicsNumber() function [46] can be applied to search for optimal number of topics from a grid of possible values. We applied the ldatuning package to the data and explored *T* = 2, 3, …, 300 topics with [21, 44, 47] metrics to determine an approximate number of topics to finally model. Note that ldatuning::FindTopicsNumber() calls topicmodels::LDA() [40] behind the scenes. However, it was not immediately clear from the package documentation whether ldatuning::FindTopicsNumber() implements a cross-validation approach which may otherwise result in a pitfall of evaluating a model on the same data it was trained on [31].

An alternative approach to determining the number of topics to model is based on *K*-fold cross-validation by splitting the data into *K* partitions, training an LDA model on the *K* − 1 partitions and using the *K*-th partition as a test data set. The model perplexity [21] is then computed on the test data set. However, cross-validation is computationally intensive especially when combined with Gibbs sampling [48].

Note that both ldatuning:FindTopicsNumber() and topicmodels::LDA() functions require the documents to be processed as a document-term matrix [34, 40]. The documents abstracts were processed into a Corpus object using the DataframeSource() function. A document-term matrix was then created by converting the Corpus text to lower case, removing standard English stop words, punctuation, numbers and stripping extra white spaces. Further, only words of at least 3 characters, occurring in at least 5 documents and at most 20% of the documents were retained. The final document-term matrix comprised of 10790 documents, 9246 terms and had a sparsity of 99%.

Finally, note that the approaches described in this section are computer intensive. Therefore, we implemented a parallel computing setup when determining the number of topics. In all cases, topicmodels::LDA() was called with *iter* = 1000, *burnin* = 100 and *seed* = 2019. The analysis was performed in an Amazon elastic compute cloud optimized instance (c5.4xlarge) [49] with 16 logical processors and 32GB of RAM, running R version 3.5.1 and RStudio version 1.1.456 [50].

## Results

### Number of topics

**Fig 1:**
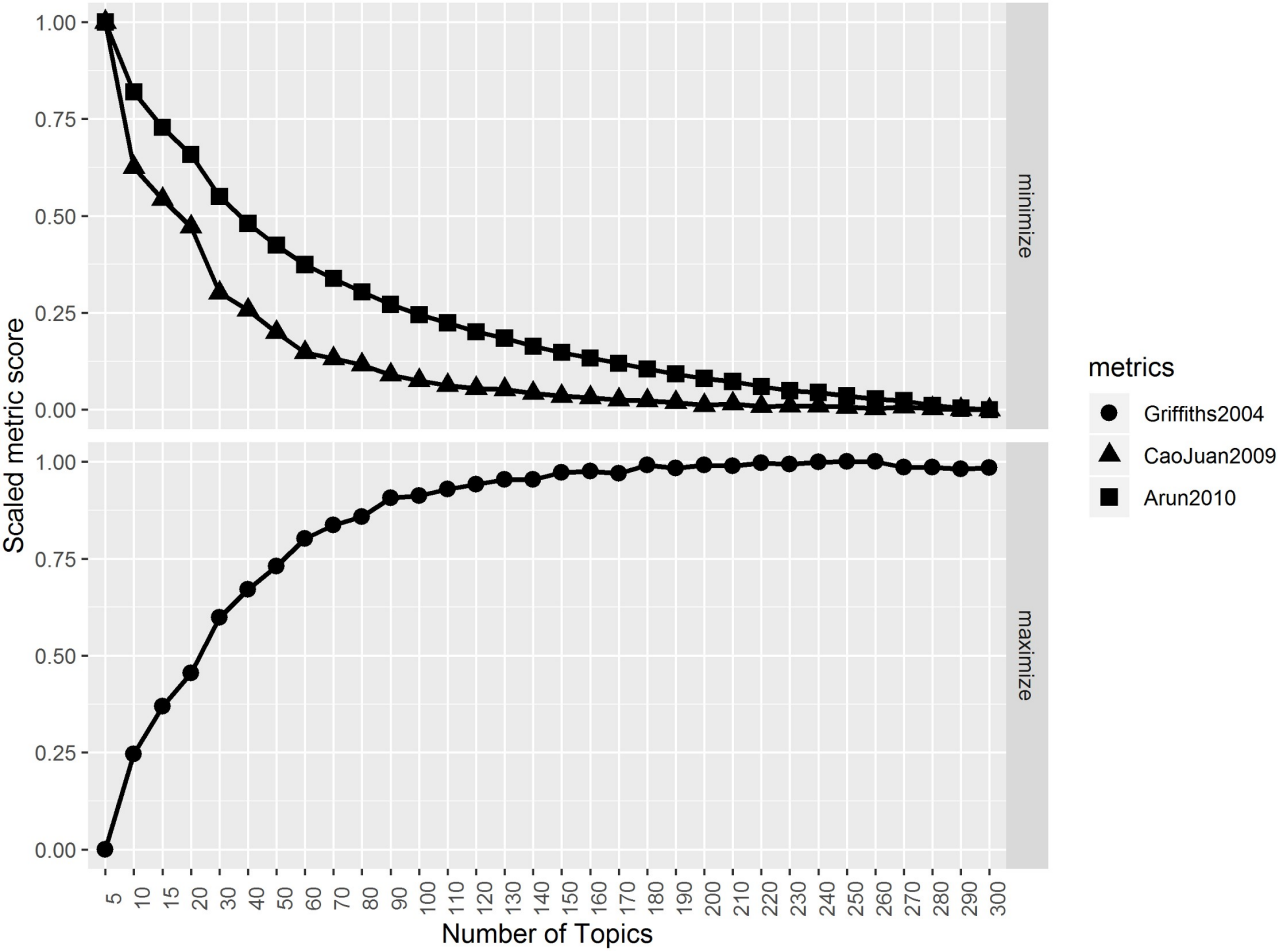
LDA metrics. Suggested number of topics using three measures as defined in ldatuning::FindTopicsNumber(). Y-axis: metric score normalized to be between zero and one. For a definition of each metric, see the corresponding reference. Upper panel: minimization-based metric smaller is better. Lower panel: maximization-based metric- larger is better.

Output of the ldatuning::FindTopicsNumber() LDA model, the results of which formed the basis for the number of LDA topics to model, is shown in Fig 1. While both [21] and [47] metrics begin to elbow-out at 60 topics and completely flatten out at around 100 topics [44], suggests a higher number of topics in general. We settled for 100 topics which was reasonable in terms of coverage and computation time.

An LDA model was fitted on the document, term matrix with 100 topics. While not large compared to the dimensions of the document-term matrix, visualization and synthesis of the results is not trivial. Therefore, we first illustrate the key output of the model fitting with the first 25 topics, a pragmatic choice to balance between readability and diversity of output, and provide the complete output of the corresponding complete 100 topics in the supplementary materials for this manuscript.

### Topics definition: Top words describing a topic

**Fig 2:**
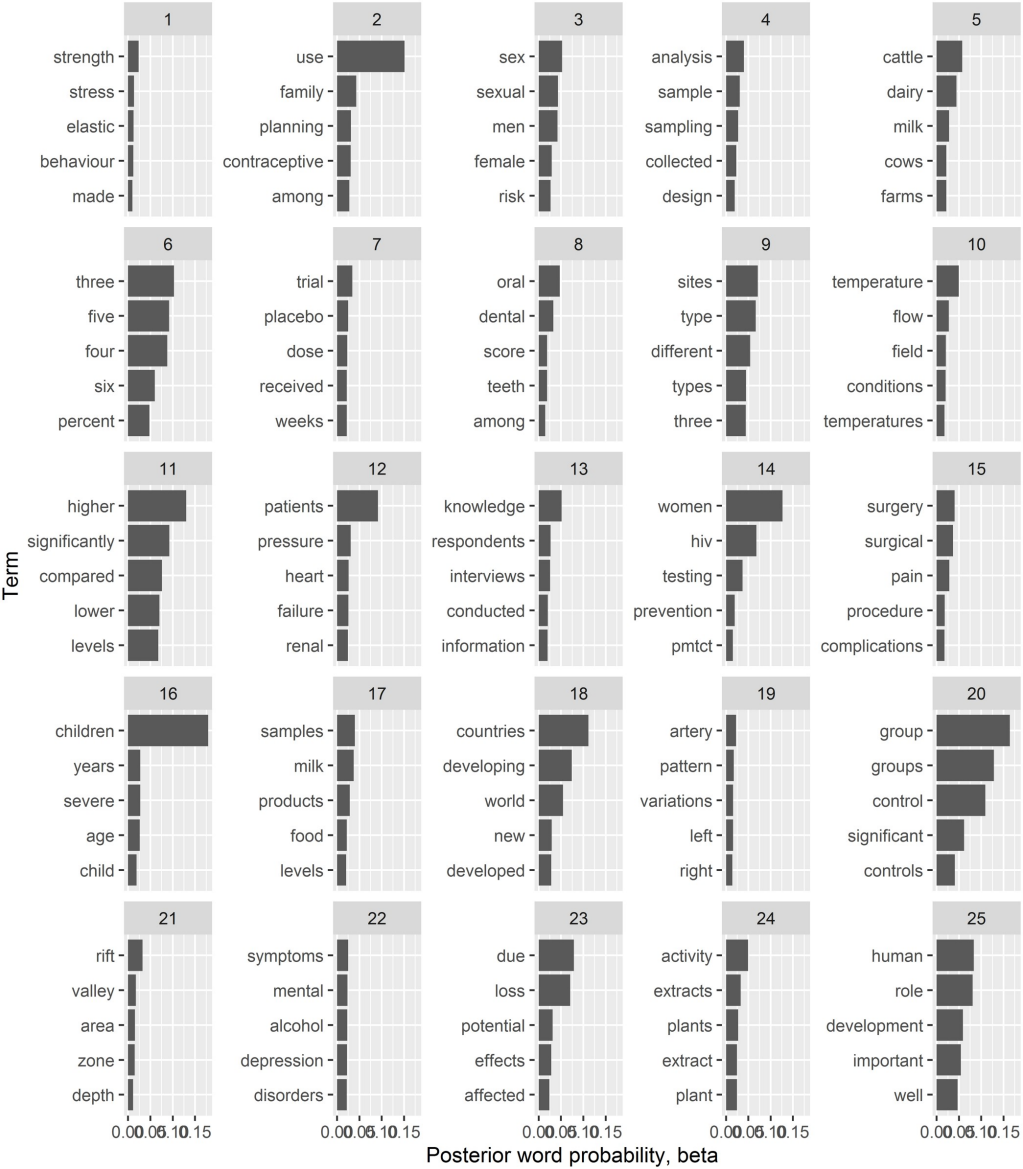
Topic themes. Posterior probability (X-axis) of the top 5 terms (Y-axis) per latent topic for the first 25 topics. The longer the bar, the more probability the corresponding term has to belong to that topic.

LDA defines a topic as a collection of co-occurring words and further, a document as a collection of topics. Fig 2 shows the posterior probability of the top 5 words defining each of the first 25 topics. While it is straight forward to assign a theme to some topics- for instance, topics 2 and 3 may be related to reproductive health, topics 4, 7 and 20 to design of clinical trials, the underlying theme may not always be obvious for some of the topics. Moreover, it is clear that several topics may be sub-themes of larger underlying themes. For instance, topics 4, 7 and 20 may be related to design of clinical trials, topics 12 and 19 involves cardiovascular health while topics 6 and 11 are comparative keywords potentially used in reporting laboratory or statistical results. This naive classification and labelling of topics has a shortcoming in that it heavily relies more on the readers understanding of the keywords given their subject matter expertise. A more comprehensive word cloud compilation of the top 100 terms defining each topic is presented in S1 Video and S1 Gif for this manuscript.

### Document classification: Posterior topic probability per document

**Fig 3:**
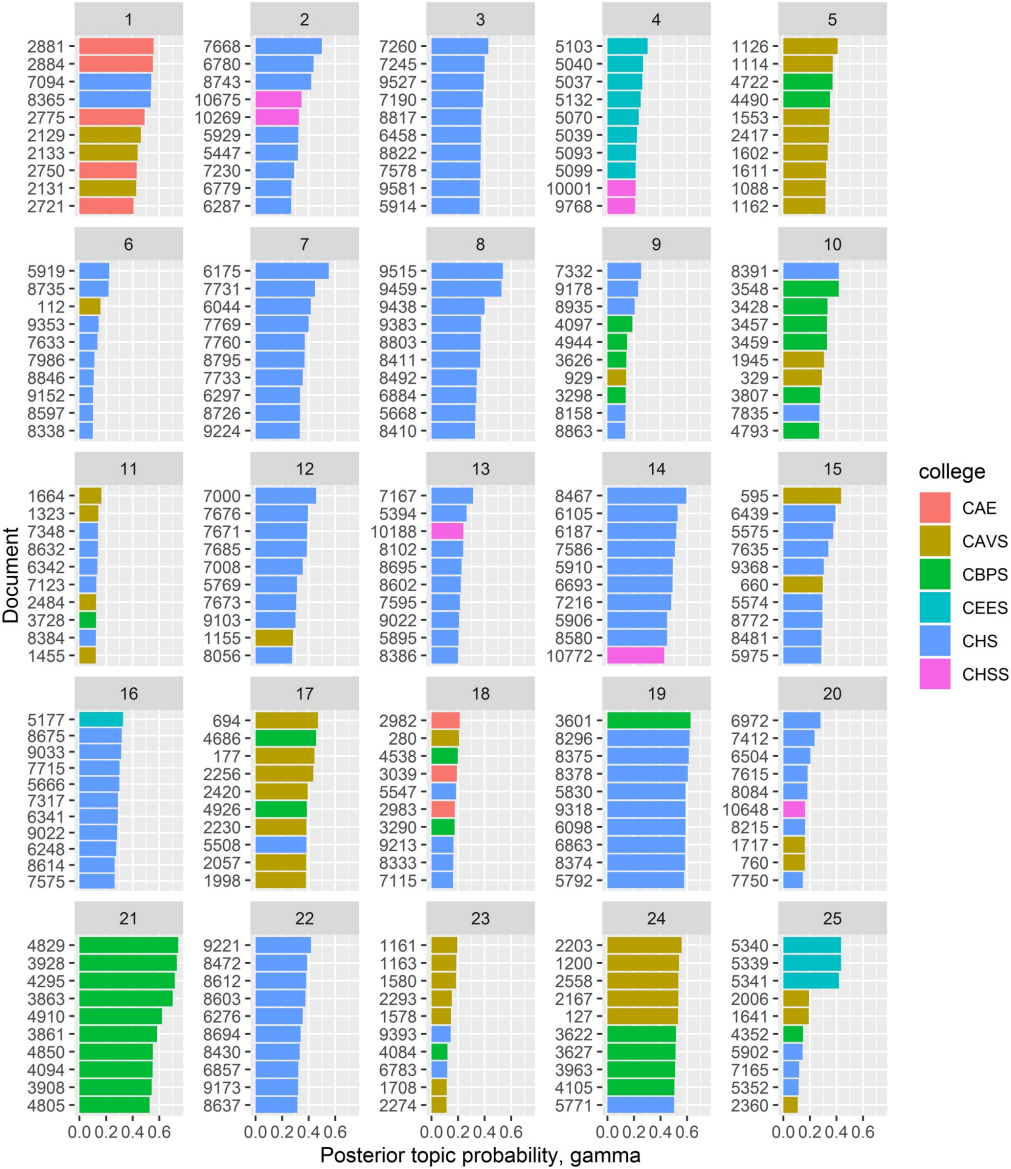
Topic themes. Posterior probability (X-axis) of the top 10 documents (Y-axis) per latent topic for the first 25 topics. Colours denote the college each document originated from.

For each topic, the posterior probability of a document given a topic is computed. In Fig 3, the posterior document probability of the top 10 documents per topic is shown for the first 25 topics. The higher the topic probability is for a document, the more likely that the topic dominates the document. Note that by definition of LDA, a document comprises of multiple topics with the topic probability being the relative composition of the topics in the document.

To link the LDA-derived topics to the natural “College” hierarchy in the data, data was obtained from six (potentially independent in terms of research themes) colleges from the University of Nairobi, we coloured the documents per topic by the college they were derived from.

A clearer association between the topics is now evident upon adding the document source label. For instance, while from Fig 2 there was no obvious association between topic 17 with both topics 18 and 21, it is now evident that the three topics are mainly derived from documents from the College of Health Sciences. The underlying theme may be related to studies designed to investigate milk and other food products in developing countries, with a focus on the Rift Valley in Africa. Another interesting example is topics 11, 19 and 25 that address specific cardiovascular sub-theme within the College of Agriculture and Veterinary Studies. However, there are topics that are strongly derived from multiple colleges such as topics 9, 13 and 15 which from the keywords, are mostly general terms that are not domain-specific.

**Fig 4:**
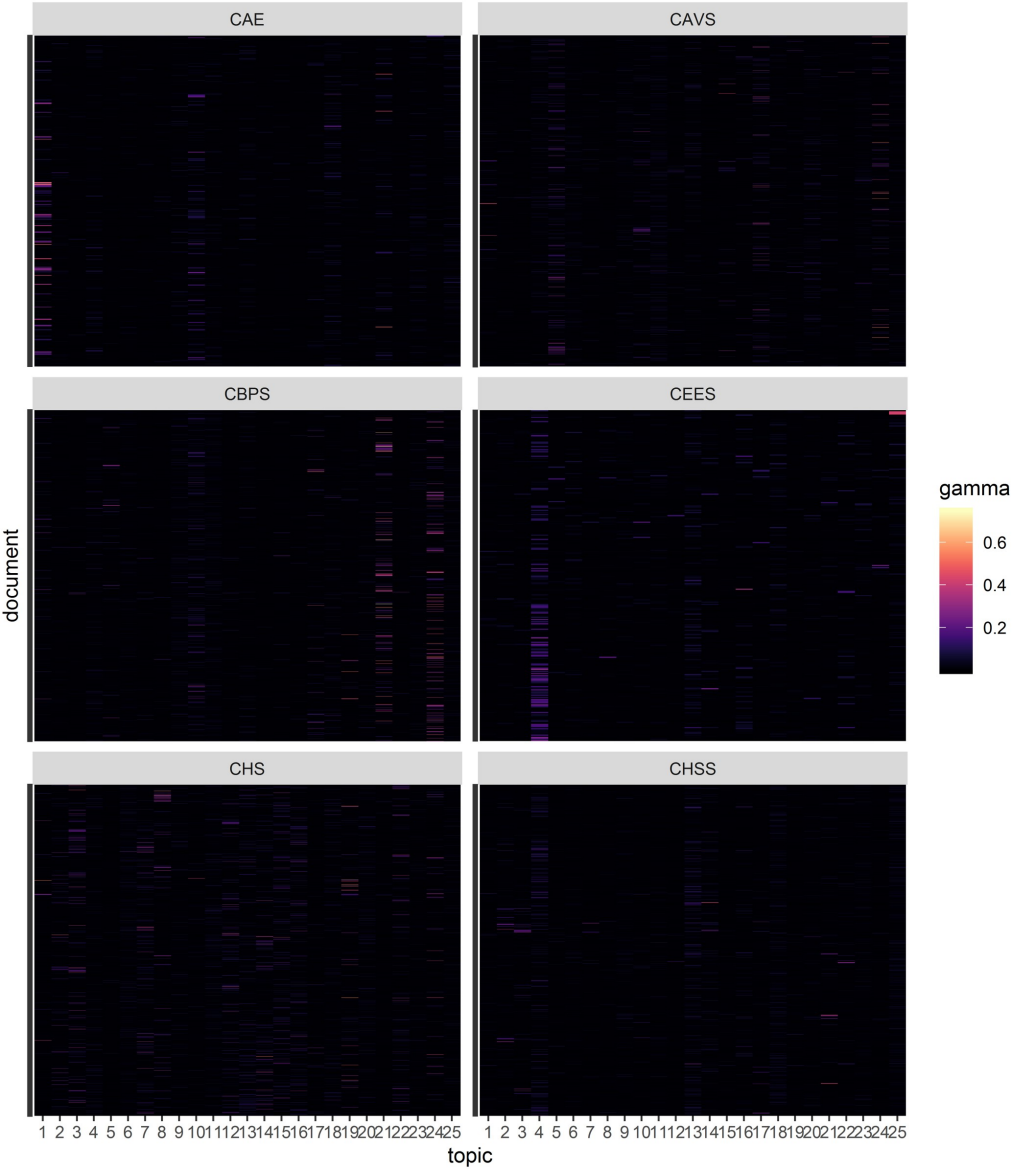
Document distribution. Posterior document probability for the first 25 topics. Y-axis: all documents from the respective college.

One feature of LDA is that each document is collection of topics whereby the probability of a document contributing to each topic is computed. In Fig 4, a raster plot of the document probability for each topic is presented. From the figure, it is clear that some topics such as topic 21 and 24 are predominantly made of specific documents from the College of Biological and Physical Sciences, while the College of Architecture and Engineering contributes strongly to topic 1. A comprehensive overview of the documents probability in all 100 topics is presented in the S1 Fig from which, it is evident that most documents are not strongly distinctively associated with one topic but weakly contribute to most topics. This can potentially be associated with a writing style that makes minimal use of domain-specific jargon, considering that the document-term matrix excluded words occurring in more than 20% of the documents. Another explanation could be that although we chose 100 topics as the optimal number of topics, this is excessive and leads to very low weights for each document across the topics.

### Hierarchical clustering of LDA-derived topics

As highlighted in the methodology section, we performed hierarchical clustering in order to reduce the number of topics into a smaller set using hierarchical clustering. We performed hierarchical clustering of the LDA document-topic posterior probability distribution matrix *γ* using the Ward.D2 agglomerative clustering algorithm [51] in R hclust().

**Fig 5:**
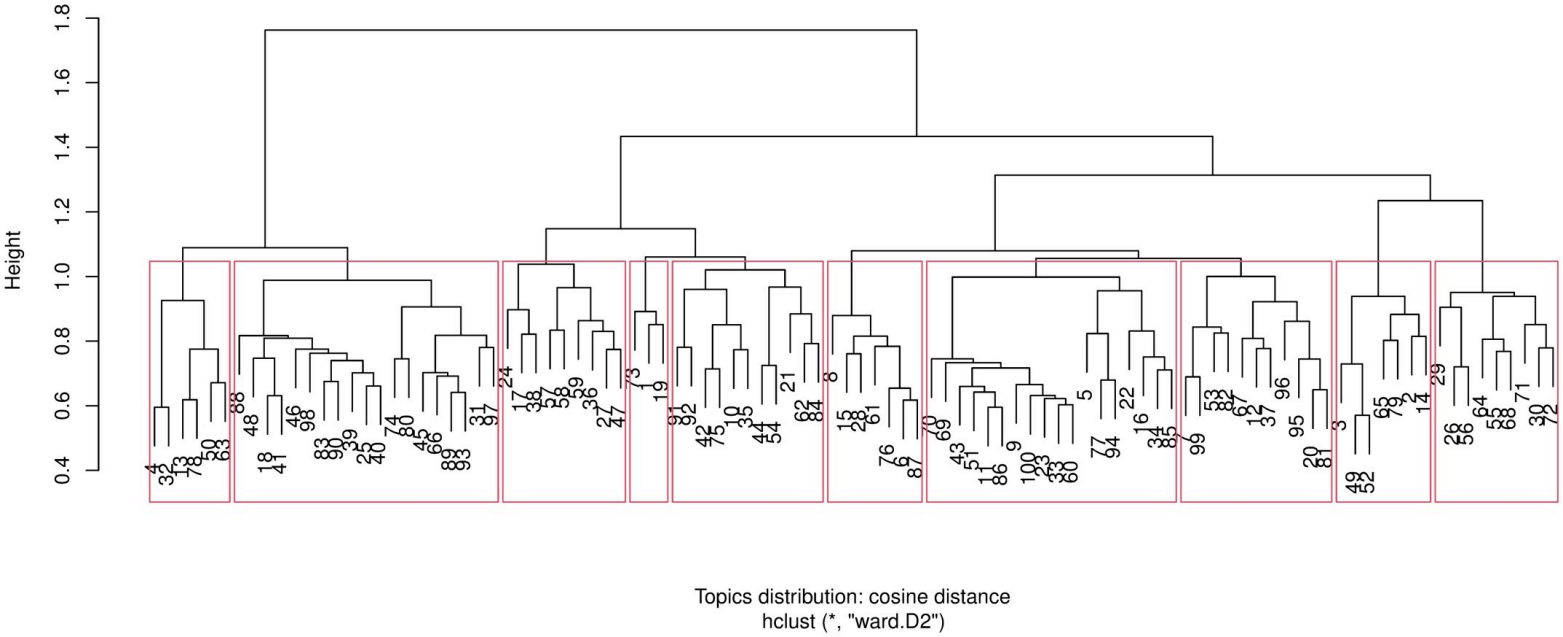
Cosine distance. Hierarchical clustering dendrogram of the 100 LDA topics. The dendrogram was cut into 10 clusters denoted by the (red) bounding rectangles. The node labels denoted the LDA topics. Y-axis: distance between the clusters as computed with Cosine distance.

**Fig 6:**
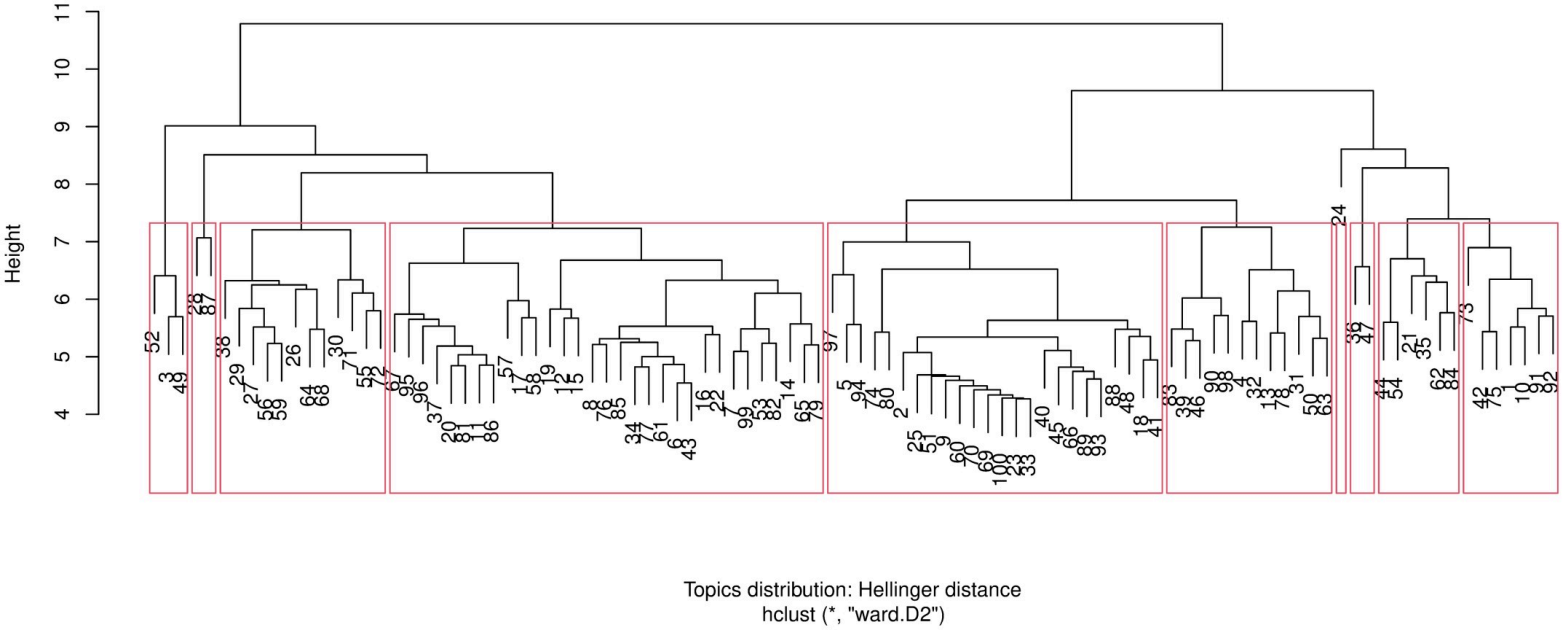
Hellinger distance. Hierarchical clustering dendrogram of the 100 LDA topics. The dendrogram was cut into 10 clusters denoted by the (red) bounding rectangles. The node labels denoted the LDA topics. The node labels denoted the LDA topics. Y-axis: distance between the clusters as computed with Hellinger distance.

The resulting dendrogram using the Cosine distance between documents in a topic is presented in Fig 5, while Fig 6 shows the dendrogram with the Hellinger distance metric. With either distance metric, a clear clustering of the 100 topics into two main branches each with sub-branches is evident.

**Fig 7:**
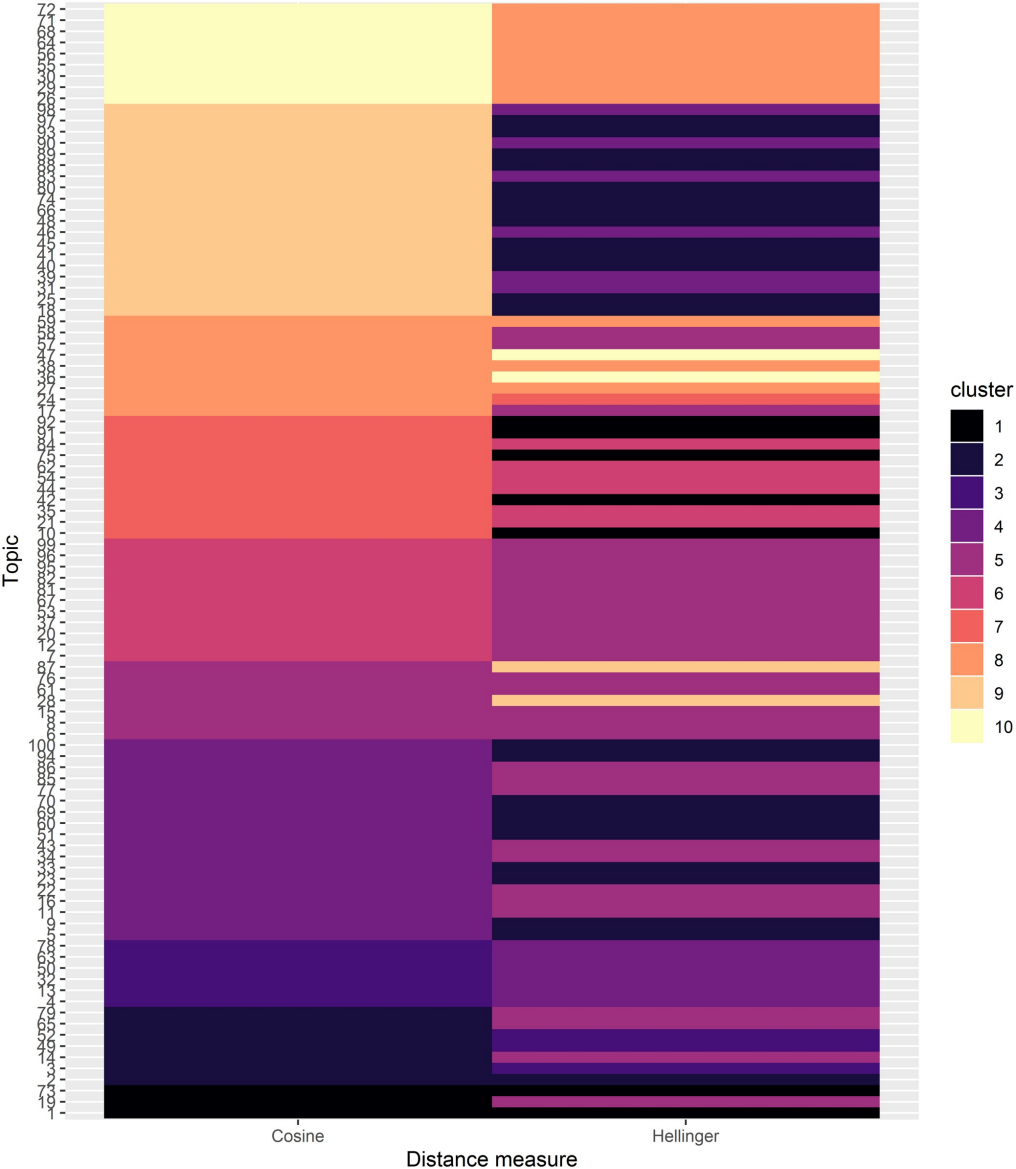
Compare distance metrics. Raster plot of the LDA topics for hierarchical clustering with two distance metrics. The trees were cut into 10 clusters and the cluster assignment for each distance metric determined. The Y-axis is ordered by Cosine distance cluster assignment. Column-wise, topics belonging to the same cluster have the same colour- although they may not be adjacent to each other as is the case with Hellinger distance column.

In Fig 7, a comparison of the Cosine and Hellinger distance-based hierarchical clustering is shown after cutting the respective dendrograms to 10 clusters (this roughly corresponds to a reasonable height at which the clusters emerge). Therefore, each cluster now comprises of several LDA topics which are similar in terms of their posterior topic probability. The topics are ordered by Cosine distance cluster assignment hence the contiguous blocks of colour for the Cosine distance column. To interprete the figure, consider the top row block of topics 72, 71, 68, 64, 56, 55, 30, 29 and 26 as a highly conserved cluster with both distance metrics while the second row block of topics 96, …, 18 are clustered together with the Cosine metric, but split into two clusters with the Hellinger distance. Overall, there doesn’t seem to be a huge disparity in terms of topic clustering with both distance metrics.

**Fig 8:**
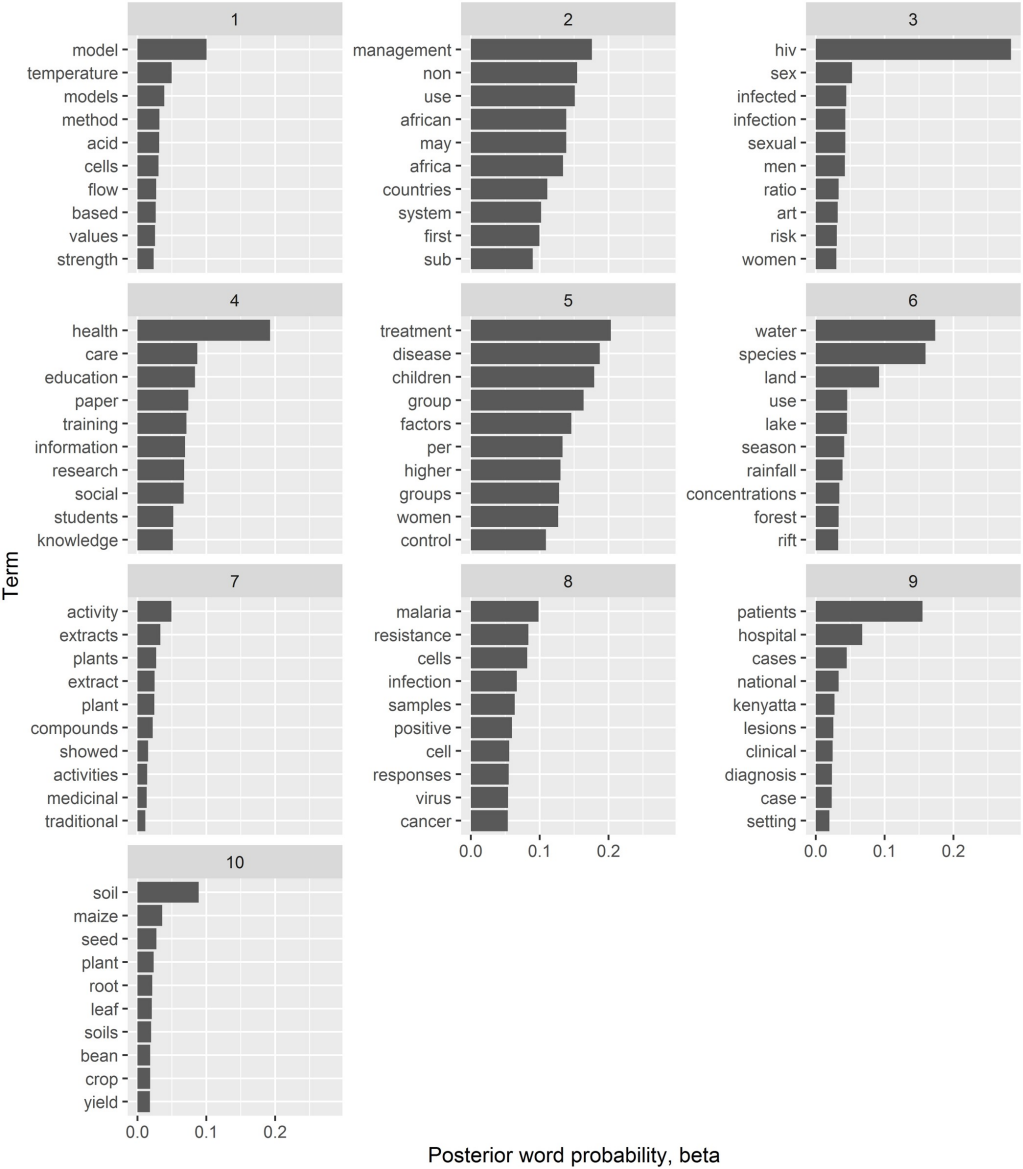
Top 10 terms. Top terms comprising the cluster of topics based on hierarchical clustering with Hellinger distance. X-axis: posterior word probability given the individual topics comprising the cluster.

**Fig 9:**
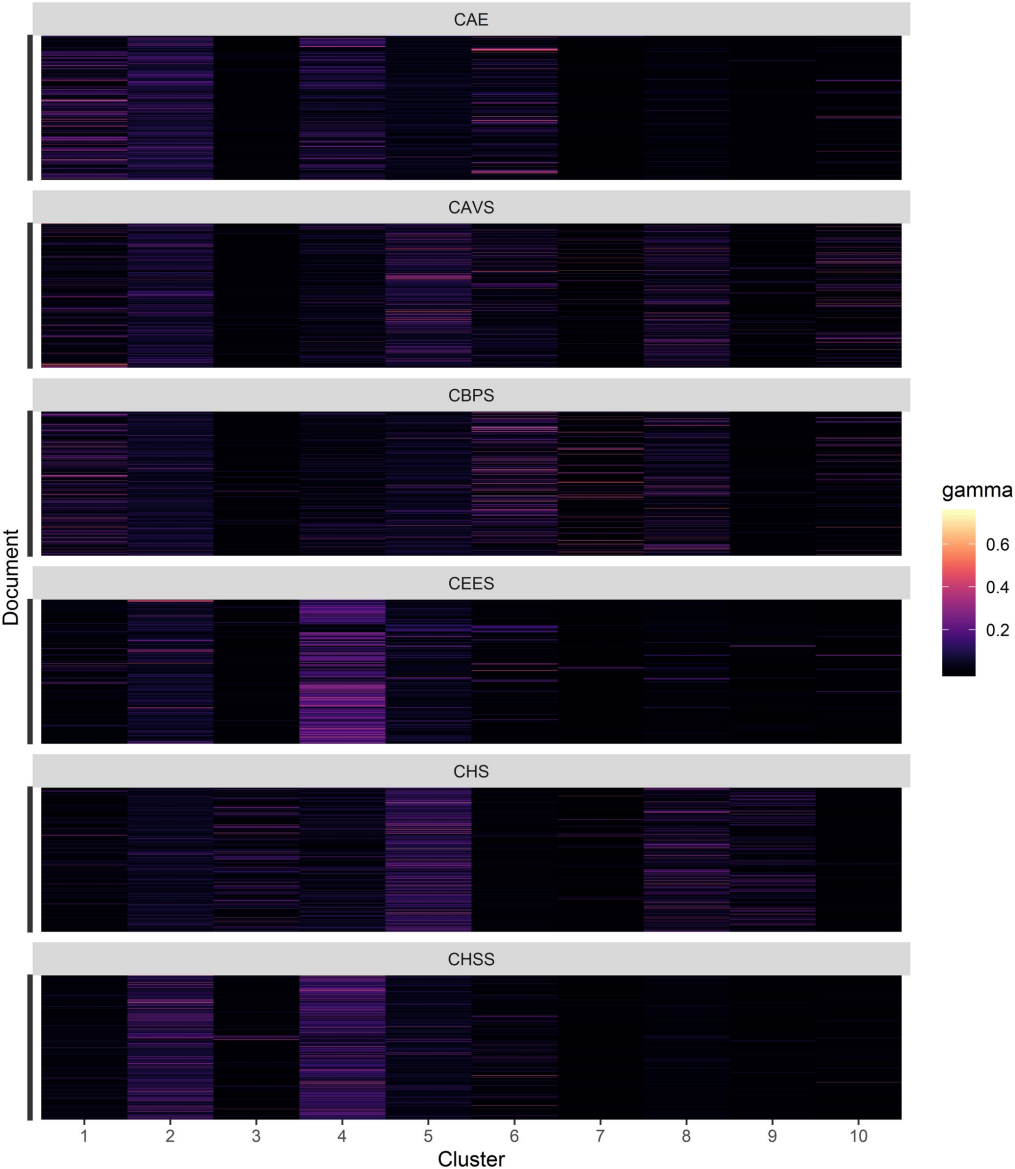
Hellinger distance. Posterior topic probability for the topic clusters. X-axis: maximum posterior probability per document for the topics comprising a cluster.

To further evaluate the results of hierarchical clustering with Hellinger distance, which collapses the 100 LDA topics to 10 thematic areas, we present the top 10 terms from the topics comprising each cluster in Fig 8, while Fig 9 shows the maximum posterior topic probability for the documents in each cluster (for each cluster, assign a document to the maximum of its topic probability, from the topics comprising the cluster).

These clusters signify a higher-level summary of the dominant research themes in the university. For instance, from Fig 8, cluster 3 refers to HIV transmission and infection dynamics in both men and women, cluster 7 seems more dedicated to topics on evaluation of plants and plant extracts for medicinal value while cluster 6 is more dedicated to research on availability and utilization of water resources.

Further, from Fig 9, it is evident that most topic clusters comprise of documents cutting across at least two colleges, other than clusters 3 and 9 which are mainly comprised of the College of Health Sciences documents- a potential indication of cross-cutting research themes amongst the colleges. For instance, cluster 4 denoting thematic areas in healthcare education (possibly community sensitization) and training (probably of social workers) seems to be a preserve of College of Education and External Studies (CEES) and College of Humanities and Social Sciences (CHSS). Another example is cluster 6 addressing various aspects of water resources availability and utilization which is a preserve of mainly the College of Agriculture and Veterinary Sciences (CAVS), College of Architecture and Engineering (CAE) and College of Biological and Physical Sciences (CBPS). The corresponding output for Cosine distance clustering are presented in the S2 and S3 Figs.

## Discussion

We applied natural language processing statistical tools to abstracts from six different colleges in the University of Nairobi. Topic modelling has proven to be a powerful tool for synthesis of large text Copora. In particular, LDA, being an unsupervised algorithm, summarizes the document text into fewer latent topics.

We have shown that while the tools are mature enough to be used with reasonable expertise, the synthesis of the results in a more succinct manner is not straightforward. Visualizations are powerful tools used to communicate results especially to non-domain experts and proper strategies to summarise results may be necessary. To this end, we applied a mix of graphical and video visualizations.

LDA, as implemented in this manuscript, assumes independence between the latent topics. On the other hand, CTM imposes a correlation between the latent topics and could potentially be applicable in the case study presented in this manuscript. However, CTM is computationally intensive and our attempts to fit a CTM model were futile due to limitations on the computing power.

As an alternative, we proposed to apply classical hierarchical clustering to document-topic probabilities matrix. As a measure of dissimilarity, appropriate distance metrics such as Hellinger and Cosine were applied. To the best of our knowledge, no previous studies have combined LDA with hierarchical clustering in this manner. In most cases, the two methods are treated independently and results compared. For instance [52], applied LDA to ecological biodiversity data and compared the results to those obtained with k-means clustering. Alternatively [53], instead applied hierarchical or K-means clustering only after further pre-processing of the LDA output to compute Euclidean distances.

From the LDA model fitting results, it was evident that some topics predominantly comprised of documents from specific colleges hence very domain specific. On the other hand, documents across different colleges contributed to several other topics. This can be attributed to use of terms in the abstracts that are not domain-specific. Further, we note that for five of the six colleges, at least two topics could be almost exclusively attributed to these colleges. This was not the case for the College of Health Sciences (CHS) whose documents were almost entirely equally distributed across the topics.

A potential explanation for this could be that research in CHS is multi-disciplinary and hence the documents text contribute to several (potentially correlated) topics. In fact, upon clustering the 100 topics into more homogeneous clusters, we note that CHS dominates clusters 3 and 9, but jointly contributes to cluster 5 with College of Agriculture and Veterinary Services (CAVS) and to cluster 8 with both CAVS and College of Physical and Biological Sciences (CPBS).

Based on the hierarchical clustering of LDA topics, it was evident that the different colleges are individually involved in at least one domain-specific research. Moreover, cross-cutting research themes were discovered especially in the case of human and animal health-related topics. As expected, most of the human health research centres around HIV and malaria (clusters 3 and 8), while agriculture (cluster 10), which is a mainstay economic activity also attract a lot of research interest.

## Conclusion

The use of open source tools for data wrangling and analysis has been illustrated with a case study comprising of a two-stage analysis of an unstructured text corpus. The hierarchical clustering approach proposed for the second stage still poses a challenge in terms of visualizing the topic clusters in relation to the LDA input documents. This is particularly the case because, while hierarchical clustering generates clusters of the original LDA topics, in the LDA step, each document may contribute to several topics. For visualization of each document within a hierarchical cluster, we used the maximum posterior topic probability across the respective topics. Thus, if hierarchical cluster one comprises of five LDA topics, the maximum of the posterior topic probability for each document is assigned as the respective posterior cluster probability.

This choice may however mask the fact that all five posterior topic probabilities for a document contributed to the formation of that hierarchical cluster. An alternative approach may be to compute a joint topic probability for each document within a hierarchical cluster (the product of the posterior topic probability for the five topics for each document). This choice has not been fully investigated and will be a subject of future improvements.

The methods described in this manuscript have many use cases in developing countries. For instance, the analysis of open-ended replies to survey questions, unstructured medical and legal documents, government social media handles and potentially for evaluating the dynamics of trending topics in the national discourse. While often written formally (in English or Swahili), more often than not, non-official social media communication- that may have relevant information in terms of trending topics- in Kenya uses slang. The methods we proposed here may potentially be used to analyse the dynamics of the language, say between urban and rural setting, across different generations or demographics or more generally, the evolution of specific text. In fact, the methodology presented in this manuscript may be applied to daily press releases by the Kenyan Ministry of Health regarding the SARS-CoV-2 outbreak. The ultimate goal would then be to evaluate the dynamic trend of the government messaging with the evolution of the pandemic.

In general, the adoption of natural language processing methods in developing countries can be hampered by the computational power required. In part, this problem can be solved by accessing cloud computing services such as Amazon Web Services, as long as a decent internet connection is available since the cloud services are web-based. However, there is still the research financing hurdle for cloud computing to be widely adopted in such countries.

## Supporting information

S1 Fig Posterior document probability for all topics. Each row denotes a document with the row×column cell representing the corresponding document probability for each topic.
(PDF)

S2 Fig Top 10 terms. Highest posterior probability of the top 10 terms comprising of clusters of topics based on hierarchical clustering with Cosine distance.
(PDF)

S3 Fig Hellinger distance. Posterior topic probability for the topic clusters. X-axis: maximum posterior probability per document for the topics comprising a cluster.
(PDF)

S1 Video Word cloud video. Top 100 terms comprising each topic. The size of the text corresponds with each term’s frequency.
(MPEG)

S1 Gif Word cloud animation. Top 100 terms comprising each topic. The size of the text corresponds with each term’s frequency.
(GIF)

S1 Data (CSV)
