# Supplementary figures and images for: Two-stage topic modelling of scientific publications: A case study of University of Nairobi, Kenya
Source: PLoS One. 2021 Jan 7;16(1):e0243208. doi: 10.1371/journal.pone.0243208 (PMC7790388; doi:10.1371/journal.pone.0243208)

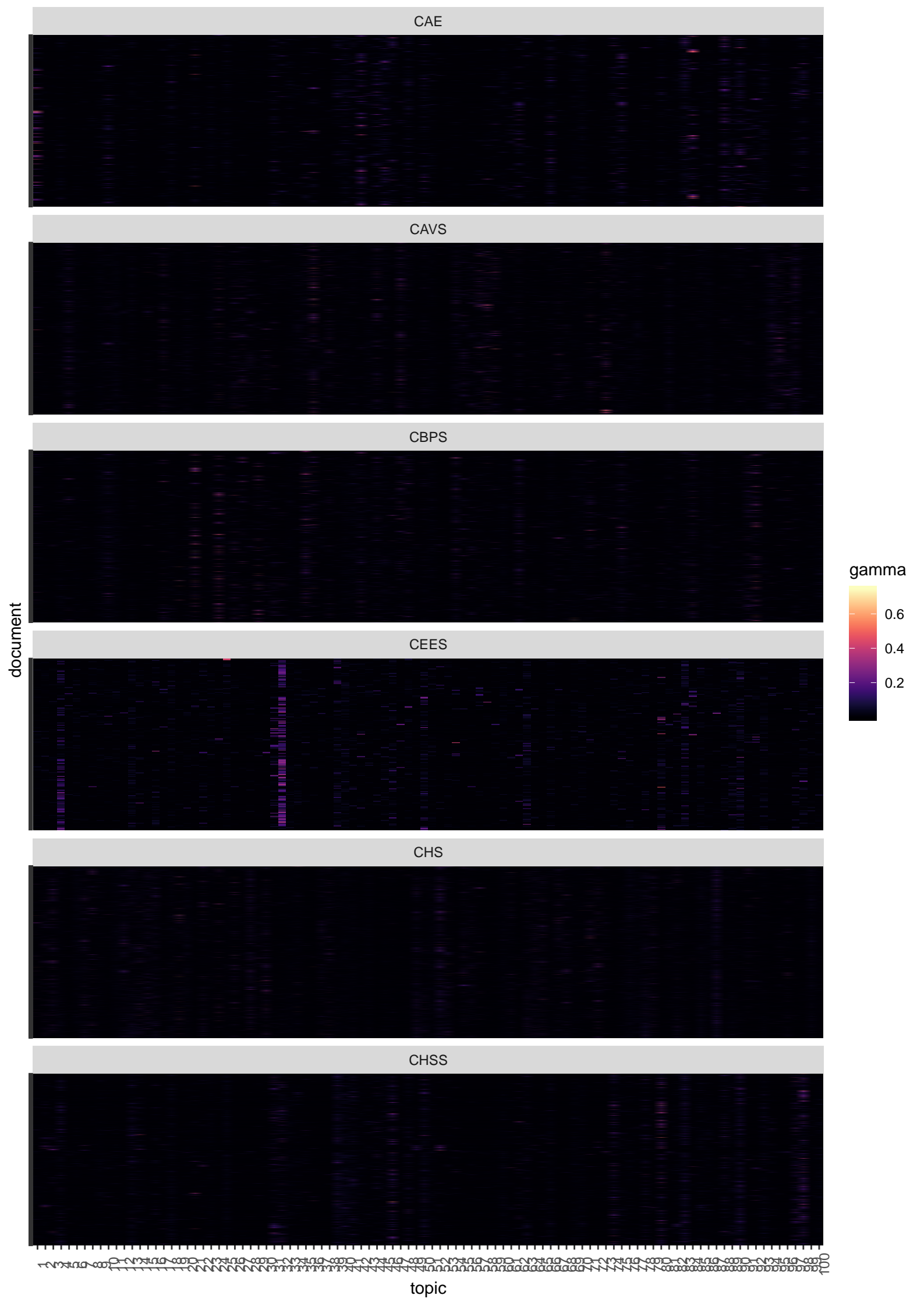

Supplement: S1 Fig — Each row denotes a document with the row×column cell representing the corresponding document probability for each topic. (PDF) [file pone.0243208.s001.pdf]

term

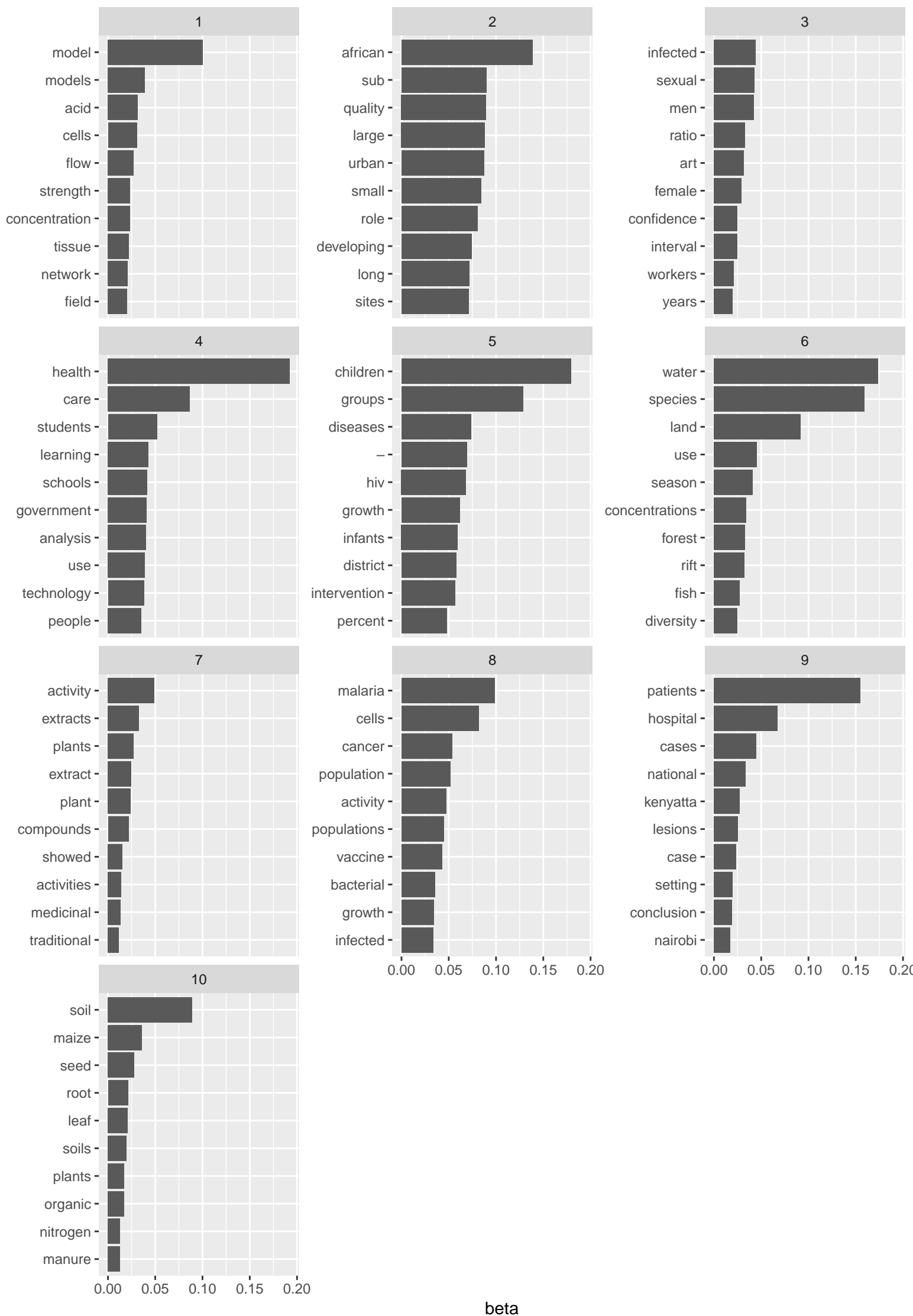

Supplement: S2 Fig — Highest posterior probability of the top 10 terms comprising of clusters of topics based on hierarchical clustering with Cosine distance. (PDF) [file pone.0243208.s002.pdf]

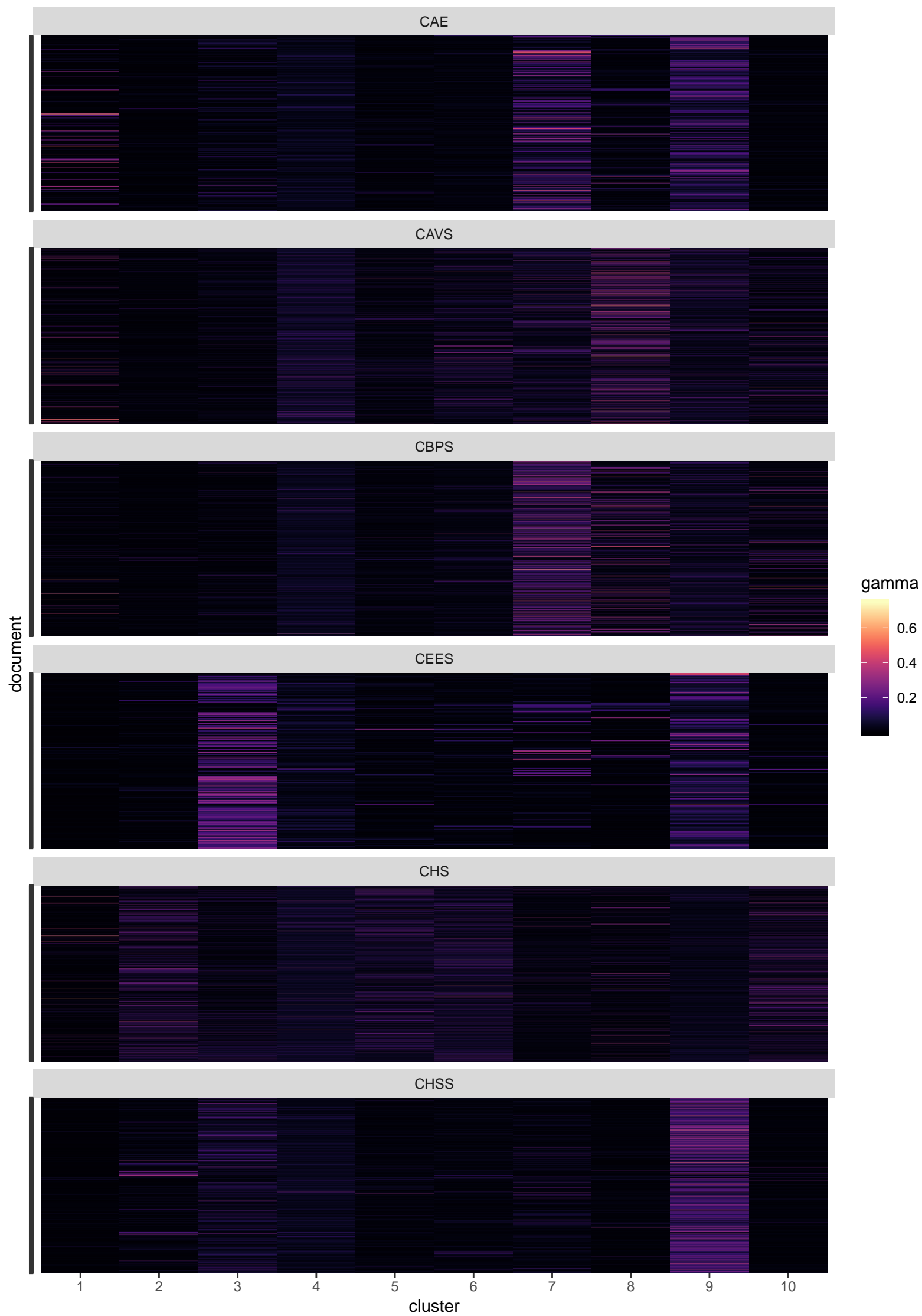

Supplement: S3 Fig — Posterior topic probability for the topic clusters. X-axis: maximum posterior probability per document for the topics comprising a cluster. (PDF) [file pone.0243208.s003.pdf]

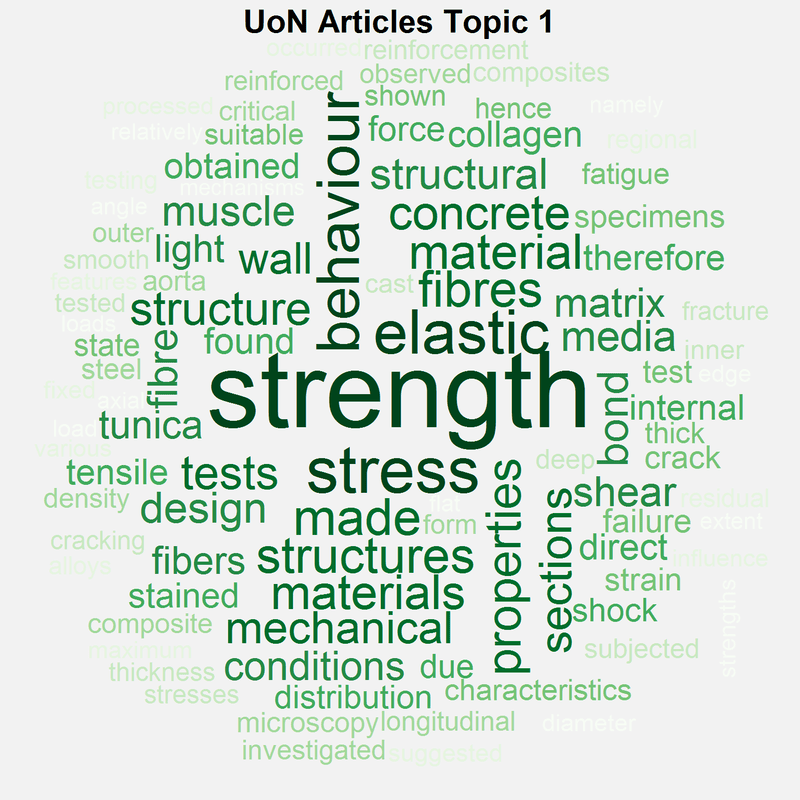

Supplement: S1 Gif — Top 100 terms comprising each topic. The size of the text corresponds with each term’s frequency. (GIF) [file pone.0243208.s005.gif]
